# Older adults have difficulty decoding emotions from the eyes, whereas easterners have difficulty decoding emotion from the mouth

**DOI:** 10.1038/s41598-022-11381-8

**Published:** 2022-05-06

**Authors:** Anna C. Y. Low, Vincent Y. S. Oh, Eddie M. W. Tong, Damian Scarf, Ted Ruffman

**Affiliations:** 1 Department of Psychology, University of Otago, P.O. Box 56, Dunedin, 9054 New Zealand; 2 Department of Psychology, National University of Singapore, Block AS4, Level 2, 9 Arts Link, Singapore, 117570 Singapore

**Keywords:** Neuroscience, Psychology

## Abstract

Older adults and Easterners have worse emotion recognition (than young adults and Westerners, respectively), but the question of *why* remains unanswered. Older adults look less at eyes, whereas Easterners look less at mouths, raising the possibility that compelling older adults to look at eyes, and Easterners to look at mouths, might improve recognition. We did this by comparing emotion recognition in 108 young adults and 109 older adults from New Zealand and Singapore in the (a) eyes on their own (b) mouth on its own or (c) full face. Older adults were worse than young adults on 4/6 emotions with the Eyes Only stimuli, but only 1/6 emotions with the Mouth Only stimuli. In contrast, Easterners were worse than Westerners on 6/6 emotions for Mouth Only and Full Face stimuli, but were equal on all six emotions for Eyes Only stimuli. These results provide a substantial leap forward because they point to the precise difficulty for older adults and Easterners. Older adults have more consistent difficulty identifying individual emotions in the eyes compared to the mouth, likely due to declining brain functioning, whereas Easterners have more consistent difficulty identifying emotions from the mouth than the eyes, likely due to inexperience inferring mouth information.

## Introduction

In this study, we examined the roles of age and culture in emotion recognition by studying young and older adults from New Zealand and Singapore. This is of interest because research indicates that (a) emotion recognition is worse in older than young adults, with the facial expressions of anger, sadness and fear creating particular difficulty^1,2^, although happiness, surprise and disgust also present some difficulty for older adults^2,3^, and (b) emotion recognition is also worse in Easterners than Westerners^4,5^. Further, there are different patterns of looking at the eyes and the mouth in young versus older adults, and Easterners versus Westerners. For this reason, we presented the eyes only, the mouth only, or the full face to participants, in order to examine whether the observed looking preferences were related to difficulty detecting emotion in particular face regions. We describe past research below, beginning with age effects.

### Age

Older adults’ greater difficulty labelling facial expressions of anger, sadness and fear is interesting because there are now many studies indicating that these three emotions are better recognized from information in the top half of the face (the eyes), in contrast to disgust and happiness, which are better recognized from the bottom half^6–13^. Interestingly, there is also evidence that, compared to young adults, older adults focus comparatively less on the eyes and more on mouths^12–17^.

Such findings have led to the suggestion that older adults’ difficulties recognizing anger, sadness and fear are due to a failure to attend to the eyes^18^. The implication is that if older adults could be encouraged to look at the eyes, then their emotion recognition should improve. We note that this argument, if correct, could not explain all emotion recognition findings. For instance, a failure to look at the eyes cannot address why older adults’ have difficulty with auditory or bodily stimuli^2,19^. Second, as discussed below, Westerners do better than Easterners on emotion recognition tasks, yet compared to Westerners, Easterners focus more exclusively on the eyes and nose, whereas Westerners look at the eyes, but also at the mouth^19–22^. Third, older adults’ failure to look at the eyes can be interpreted as a *cause* of worse recognition of anger, sadness and fear as hypothesized, but equally, their difficulty understanding eyes information might *cause* them to look less at the eyes. If less eyes looking causes worse emotion recognition, then presenting the eyes alone should assist recognition because there is nothing else to look at (i.e., there is no mouth or other facial features to distract attention). If, in contrast, older adults look less at the eyes because they struggle to interpret eyes information, then presenting the eyes alone will not help recognition and they will still be worse than young adults.

To examine the role of eyes looking in recognition, previous researchers have computed correlations between eye gaze and recognition. For instance, one study found that more looking at the upper face correlated with better recognition of anger, fear and sadness in young and older adults, although there was no relation between *mouth* looking and emotion recognition^13^. In contrast, two other studies found no relation between either eyes looking or mouth looking and emotion recognition^12,17^. Overall, then, there is no clear pattern as to whether looking correlates with recognition. Indeed, it is likely that looking at the eyes or mouth fails to correlate with emotion recognition because one can posit either a positive or negative correlation. For instance, if looking at the eyes *helps* recognition of anger, sadness and fear, then one would expect a positive correlation. However, if one looks more at the eyes because of *difficulty* recognizing anger, sadness and fear, then one would expect a negative correlation. These different relations could be manifest in different participants, so that when one looks at the group as a whole these two things would balance each other out, leaving no clear pattern. Therefore, rather than examining correlations, a clearer method that we used to examine whether older adults have particular difficulties interpreting eyes information is to split the face into eyes only or mouth only.

### Culture

People are often better at recognizing emotions within their ethnic group^23^. However, even when a mixture of White and Asian/Japanese faces are used, Japanese and Chinese individuals do not label facial expressions in the same way as Westerners, with performance worse in Eastern individuals^24,25^. Easterners and Westerners seem to use different cues. For instance, Westerners associate a distinct set of facial features with different emotions (sometimes the eyes, sometimes the mouth, sometimes the eyebrows and mouth), but Easterners tend to consistently use the eyes to identify each emotion^26^.

There are also differences in the face regions that Easterners and Westerners look at. For instance, Westerners fixated predominately on the eyes when identifying emotion in faces, but also on the nose and mouth^27^. In contrast, it is thought that Easterners may avoid looking at the eyes because direct eye contact is considered rude^28^. To this end, young Chinese adults look more at the lower part of emotion faces (i.e., which included the nose and mouth) than the upper part (i.e., the eyes)^29^. However, there are also contradictory findings in that Malaysian Chinese tend to look most at the eyes when given emotion faces, then the nose, and then the mouth^30^. Nevertheless, these studies are hard to interpret because (a) the results were contradictory and (b) each study tested only Westerners or Easterners (but not both), so it is possible that idiosyncrasies in the stimuli in different studies caused the differences. Thus, a better test of Eastern-Western differences is to compare the two groups using the same set of stimuli.

**Figure 1:**
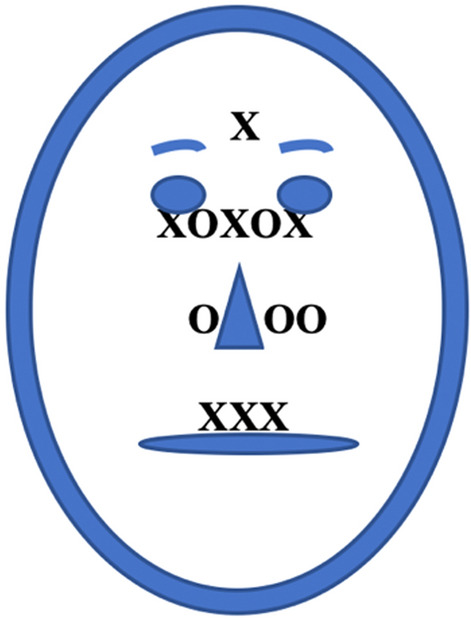
Number of prior studies indicating gaze tendencies at particular face regions in Easterners and Westerners. Note. X: Westerners, O: Easterners. For example, three prior studies indicate substantial looking at the nose by Easterners, whereas three studies indicate substantial looking at the mouth by Westerners. The figure shows that looking is more widely distributed across the face for Westerners compared to Easterners.

Several studies have directly compared Easterners and Westerners using the same set of stimuli. In one study, Westerners looked more at the eyes, whereas Easterners looked more at the nose of an emotionally neutral face^31^. Other studies found that Westerners looked at the eyes and mouth of emotional faces, whereas Easterners looked more centrally at the nose^5^, Westerners looked at the eyes and mouth whereas Easterners looked at the eyes and nose^21^, and Westerners looked more at the eyebrows and mouth compared to Easterners, who looked more at the eyes on their own^26^. These four studies directly comparing Easterners and Westerners all used both Eastern and Western still-face stimuli so there was no ethnic bias in the stimuli themselves. The participants’ looking tendencies are displayed in Fig. 1, which shows that Westerners tend to have a more distributed gaze pattern (taking in both higher and lower face regions and avoiding the very middle nose region), whereas Easterners’ looking tends to be more centralized to the eyes and nose.

Besides looking predominately at the eyes (and nose) there is also evidence that Easterners might rely more on the eyes to interpret facial expressions, whereas young Westerners rely more on the eyebrows and mouth^26,32^. For instance, one study manipulated the mouth and eyes of Western faces, creating emotion hybrids (e.g., happy eyes with sad mouth; sad eyes with happy mouth)^32^. They found that Japanese individuals tended to use the eyes more than the mouth whereas Western individuals tended to use the mouth more than the eyes. The authors argue that the mouth is more emotionally expressive than the eyes, but in Eastern cultures, expressions are usually concealed, sometimes by directly obscuring the mouth with a hand. Thus, Easterners benefit from the eyes because they are used to emotions being concealed and unused to full expression in the mouth, whereas Westerners benefit from the mouth because of greater expression of emotion in the mouth in the West and less of a tendency toward concealment. In sum, the results show a different bias to use eyes versus mouth in the two cultures. Yet, because there was no “correct” answer as to what emotion was actually expressed, the results don’t reveal whether each culture *can* correctly use eyes or mouth to infer emotion. In our study, when presenting the eyes or mouth on their own, there was a correct answer as to what emotion was expressed, so that we could gauge emotion recognition success with eyes-only or mouth-only stimuli.

In sum, there are age group and cultural differences in emotion recognition, and in the face regions individuals in these groups are purported to use to infer emotion. Nevertheless, these studies simply measured looking at eyes or mouth, or the bias to use the eyes or mouth to infer emotion, but there was no way of examining whether participants could independently use each region to correctly infer emotion. In our study, we presented just the eyes or just the mouth to examine this question.

### Present study

We examined facial emotion recognition in a group of young and older adults from the West (New Zealand) and the East (Singapore), with a large set of emotion stimuli (108 items) split into eyes only, mouth only or full face. On the basis of previous findings, we expected young adults to be better than older adults with eyes information, and Easterners to be better with eyes information than mouth information. In general, we expected older adults to be better with mouths than eyes, and Westerners to be better with mouths than Easterners.

## Results

First, we directly compared Eastern and Western participants’ emotion recognition on the Asian-Chinese versus the European emotion photos. Then, we pooled the stimuli to examine general emotion recognition in young versus older adults, and Eastern versus Western individuals.

### Comparison of Asian versus European stimuli

In a preliminary analysis, we examined whether the unbiased hit rates for Westerners and Easterners were different for same-race versus other-race stimuli using a 2 (Participant Ethnicity) × 2 (Stimuli Ethnicity) analysis of variance (ANOVA). There was a main effect for Stimuli Ethnicity, *F*(1, 215) = 7.59, *p* = 0.006, *η*_*p*_^2^ = 0.034, a main effect for Participant Ethnicity, *F*(1, 215) = 133.68, *p* < 0.001, *η*_*p*_^2^ = 0.383, and a significant interaction, *F*(1, 215) = 43.90, *p* < 0.001, *η*_*p*_^2^ = 0.170. The interaction was examined with two paired-samples *t* tests. Easterners were better with Eastern stimuli (*M* = 0.425, *SD* = 0.138) than Western stimuli (*M* = 0.328, *SD* = 0.275), *t*(106) = 5.61, *p* < 0.001, *d* = 0.796. Conversely, Westerners were better with Western stimuli (*M* = 0.639, *SD* = 0.119) than Eastern stimuli (*M* = 0.599, *SD* = 0.102), *t*(109) = 3.47, *p* < 0.001, *d* = 0.333. Comparisons between studies in absolute success rates are difficult because researchers use different emotion expressions (e.g., just anger and happiness in some studies versus all six basic emotions in other studies), and because they report different outcome measures (e.g., d′, *Hu*, proportion correct, or use radar charts). Although an advantage on same-race stimuli is not always obtained^33,34^, our finding of better emotion recognition with same-race stimuli is consistent with many previous studies^35^, and therefore, provides reassurance that the stimuli acted as expected. However, because our primary interest was in emotion recognition with a set of stimuli that was not biased toward one group or the other, the subsequent analyses below utilized the pooled stimuli set.

### Comparisons of age and culture

**Figure 2:**
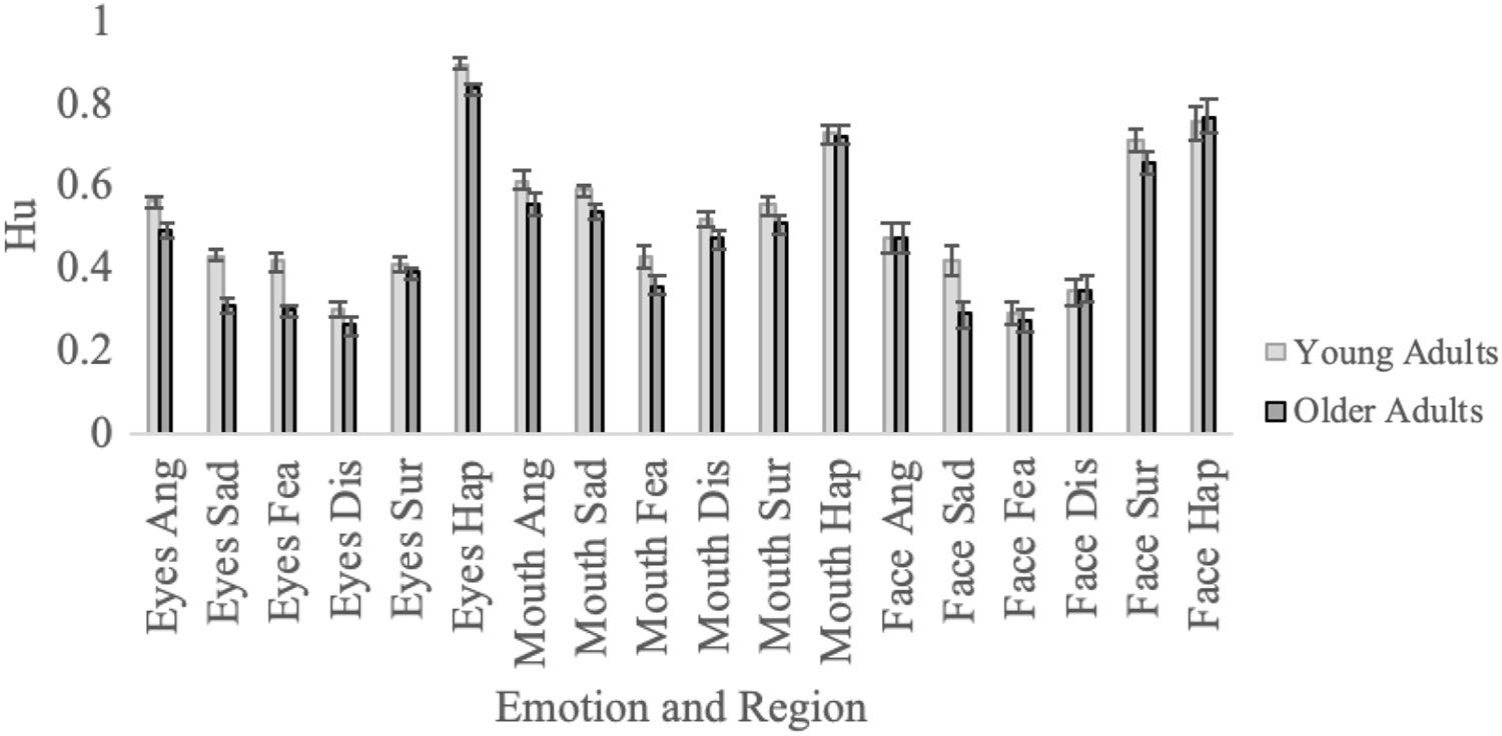
Young and older adults’ emotion recognition when shown the eyes, mouth or full face. Note. Bars represent standard errors. Relative to young adults, older adults had more consistent difficulty on the Eyes Only stimuli than the Mouth Only stimuli. For the Eyes Only stimuli, older adults were worse than young adults on anger, sadness, fear and happiness. For the Mouth Only stimuli, older adults were worse only on sadness.

**Figure 3:**
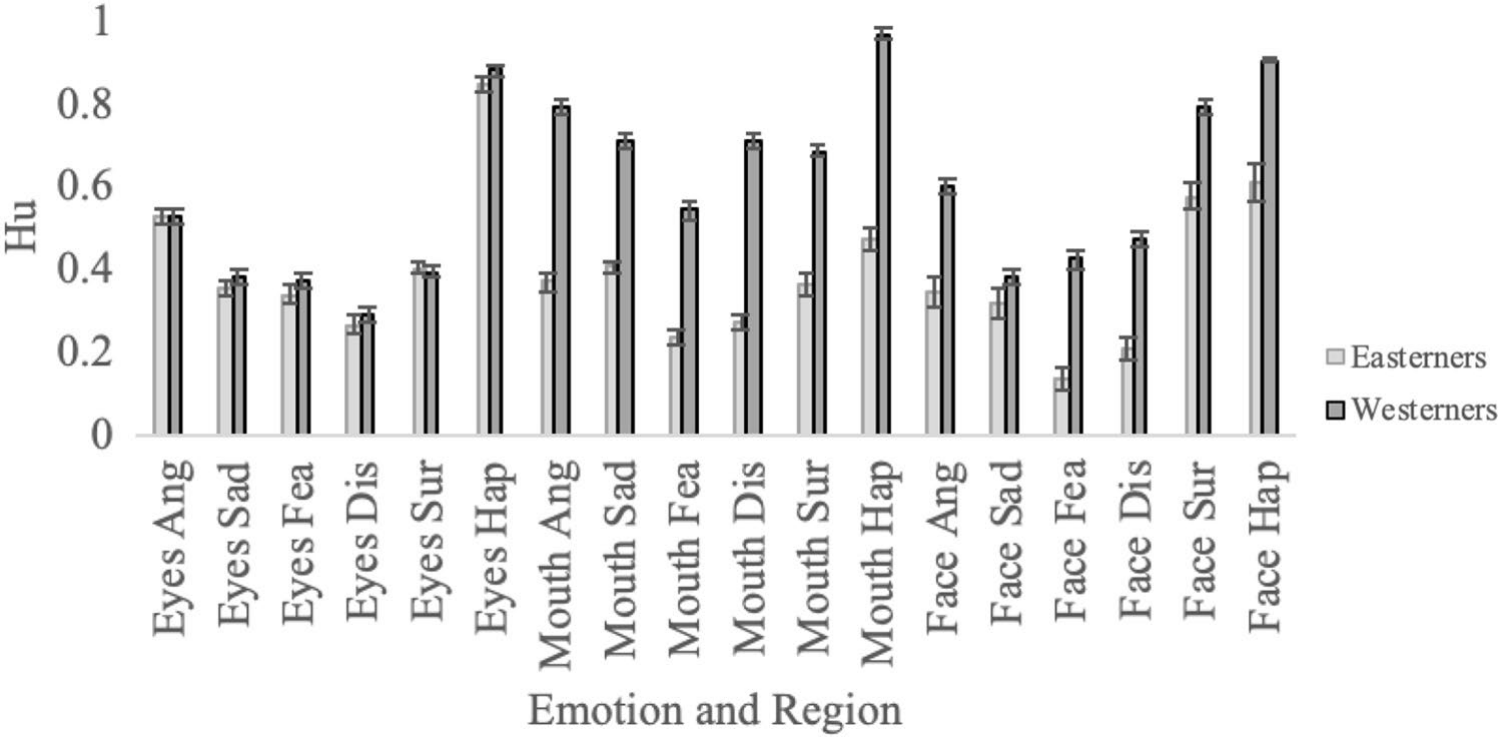
Easterners’ and Westerners’ emotion recognition when shown the eyes, mouth or full face. Note. Bars represent standard errors. There was no difference in the emotion recognition of Easterners and Westerners on the Eyes Only stimuli. In contrast, Easterners were worse on all six emotions of the Mouth Only stimuli and the same was true for the Full Face stimuli.

Figures 2 and 3 include the unbiased hit rates (*Hu*) for emotion recognition, broken down according to participant age group and ethnicity, and face region and emotion. The data were analyzed with a 2 (Participant Age Group: young, old) × 2 (Participant Ethnicity: Westerners, Easterners) × 3 (Face Region: Eyes Only, Mouth Only, Full Face) × 6 (Emotion: anger, sadness, fear, disgust, surprise, happiness) ANOVA, with the first two variables between-subjects and the last two variables within-subjects. The dependent variable was the mean unbiased hit rate (*Hu*) for emotion recognition. A summary of the effects from the ANOVA is included in Table 1, with significant effects shown in bold font.

**Table 1:** Summary of emotion recognition effects from the analysis of variance.

	*F*	*p*	*η*_*p*_^2^
**Face Region**	**20.05**	**< 0.001**	**0.086**
Face Region × Participant Age Group	1.10	0.332	0.005
**Face Region** **×** **Participant Ethnicity**	**95.64**	**< 0.001**	**0.310**
Face Region × Participant Age Group × Participant Ethnicity	0.45	0.639	0.002
**Emotion**	**552.19**	**< 0.001**	**0.722**
**Emotion** × **Participant Age Group**	**5.27**	**< 0.001**	**0.024**
**Emotion** **×** **Participant Ethnicity**	**13.26**	**< 0.001**	**0.059**
Emotion × Participant Age Group × Participant Ethnicity	1.12	0.347	0.005
**Face Region** **×** **Emotion**	**96.60**	**< 0.001**	**0.312**
**Face Region** **×** **Emotion** **×** **Participant Age Group**	**3.04**	**0.002**	**0.014**
**Face Region** **×** **Emotion** **×** **Participant Ethnicity**	**8.68**	**< 0.001**	**0.039**
Face Region × Emotion × Participant Age Group × Participant Ethnicity	1.43	0.176	0.007
**Participant Age Group**	**7.63**	**0.006**	**0.035**
**Participant Ethnicity**	**123.29**	**< 0.001**	**0.367**
Participant Age Group × Participant Ethnicity	0.01	0.971	0.000

Significant effects are in bold text.

The main effect for Face Region was explored with three paired-samples *t* tests, indicating better performance on the Full Face stimuli (*M* = 0.748, *SD* = 0.118) than the Eyes Only stimuli (*M* = 0.464, *SD* = 0.110), *t*(216) = 37.29, *p* < 0.001, *d* = 2.54, and the Mouth Only stimuli (*M* = 0.587, *SD* = 0.113), *t*(216) = 14.86, *p* < 0.001, *d* = 1.39. In addition, performance on the Mouth Only stimuli was better than on the Eyes Only stimuli, *t*(216) = 20.33, *p* < 0.001, *d* = 1.02. The main effect for Emotion was not of theoretical interest and so was not explored further. The main effect for Participant Ethnicity indicated that Westerners (*M* = 0.619, *SD* = 0.093) were more accurate than Easterners (*M* = 0.377, *SD* = 0.198). The main effect for Age Group indicated that young adults (*M* = 0.490, *SD* = 0.207) had better emotion recognition than older adults (*M* = 0.443, *SD* = 0.202).

There were six significant interactions, including two three-ways and four two-ways. Because all of the two-way interactions were subsumed by the three-ways, we only explored the three-way interactions. The three-way interaction between Face Region, Emotion and Participant Age Group is shown in Fig. 2 and was examined with three 2 (Age Group) × 6 (Emotion) ANOVAs, one for each face region. Of interest was the two-way interaction between Age Group and Emotion, which was significant for Eyes Only, *F*(5, 1075) = 3.88, *p* = 0.002, *η*_*p*_^2^ = 0.018, and Mouths Only stimuli, *F*(5, 1075) = 6.22, *p* < 0.001, *η*_*p*_^2^ = 0.028, but not for the Full Face stimuli, *F*(5, 1075) = 1.28, *p* < 0.269, *η*_*p*_^2^ = 0.006. The significant interactions indicate that, with Eyes Only or Mouth Only stimuli, older adults found some emotions particularly difficult compared to young adults. We examined these effects further with independent samples *t* tests. For the Eyes Only stimuli, older adults were worse on all emotions, all *t*s > 2.90, all *p*s < 0.005, all *d*s > 0.388, except disgust and surprise, both *t*s < 1.01, both *p*s > 0.317. For the Mouth Only stimuli, older adults were only worse on sadness, *t*(215) = 5.55, all *p* < 0.001, *d* = 0.754, but not any of the other emotions, all *t*s < 1.53, all *p*s > 0.128. While indicating that older adults have some difficulty deciphering emotion information in the mouth, these findings provide important information that the more consistent source of older adults’ difficulties on individual emotions is the eyes.

The three-way interaction between Face Region, Emotion and Participant Ethnicity is shown in Fig. 3 and was examined with six 2 (Participant Ethnicity) × 3 (Region) ANOVAs, one for each emotion. Of interest was the interaction between Participant Ethnicity and Face Region. This was significant for all emotions, all *F*s > 28.25, all *p*s < 0.001. These interactions indicate that compared to Westerners, Easterners found emotions harder to detect from some regions than others. To explore the interactions further we compared Easterners’ and Westerners’ looking using independent samples *t* tests for each emotion in each of the three face regions. We treated each region as a family of comparisons (three families in total), and used Holm’s correction to ensure that the family-wise error rate was kept to *p* < 0.05. There was no difference in Easterners’ and Westerners’ emotion recognition on the Eyes Only stimuli for any of the six emotions, all *t*s < 1.40, all *p*s > 0.164. In contrast, Westerners’ emotion recognition was better than that of Easterners for all six emotions for the Mouths Only stimuli, all *t*s > 2.48, all *p*s < 0.015, and the same was true for full faces, all *t*s > 7.49, all *p*s < 0.001. This is a striking difference in difficulty for the different face regions.

Importantly, although older adults had difficulty on the Eyes Only stimuli and Easterners had difficulty on the Mouth Only stimuli, valid emotional cues *were present*. For instance, both young and older adults were above chance on all emotions for the Eyes Only and Mouth Only stimuli, all *p*s < 0.001, even after correcting for multiple comparisons. Similarly, Westerners were well above chance on all emotions for the Eyes Only and Mouth only stimuli, all *p*s < 0.001, and Easterners were above chance on all emotions (all *p*s < 0.001 after correcting for multiple comparisons) except fearful and disgusted mouths. These results show that there were valid emotional cues in the stimuli but that older adults and Easterners were worse at discerning these clues.

Finally, we present the confusion matrices in the Supplementary Materials (Tables S1 and S2), detailing errors for the full-face stimuli. The final two rows of each table indicate the percentage of errors that were angry, sad, fearful, disgusted, surprised or happy for each group. The errors were, for the most part, remarkably similar when comparing young to older adults, and were also broadly similar for Easterners versus Westerners.

## Discussion

In our study we presented only the eyes, only the mouth, or the full face. This allowed us to examine whether young and older individuals from New Zealand and Singapore had genuine difficulty identifying emotion in these face regions. We summarize our results for age group and culture separately below.

### Age group

On the one hand, there was not an interaction between age group and face region, which indicates older adults’ *overall* performance on the eyes versus the mouth was not different to that of young adults. However, older adults’ difficulty with mouth stimuli was restricted to a single emotion: sadness. This was the only emotion older adults were worse identifying from the mouth compared to young adults. In contrast, they had more consistent difficulty identifying individual emotions from the eyes, including anger, sadness, fear and happiness.

Our results are consistent with previous findings that older adults find anger, sadness and fear difficult to identify^2,3^. Further, they help to explain *why* older adults look comparatively less at the eyes than young adults^12,13,17,19^. They likely look less because they have consistent difficulty discerning emotional information in the eyes (resulting in worse performance than young adults on 4 of 6 emotions when presented with the eyes only). Conversely, they likely look more at mouths than young adults because their difficulties decoding mouth information are more isolated (with worse performance relative to young adults only on sadness, and equivalent performance on 5 of 6 emotions).

Recall, also, that previous studies examining correlations between older adults’ eyes looking and emotion recognition were inconsistent^12,13,17^. We argued that this was because more eyes looking could either assist emotion recognition, or indicate difficulty decoding eyes information. The results of the present study suggest that difficulty better explains older adults’ looking patterns. Further, recall the argument that older adults’ emotion recognition difficulties stem from a failure to “attend to the eyes”^18^. In the Eyes Only condition there was nowhere else on the face for participants to look; participants could not look at mouths and could only attend to the eyes, yet this clearly did not relieve older adults’ of their difficulties. That they continued to have difficulty despite being compelled to look at the eyes, indicates that older adults’ difficulty was not due to a failure to look at the eyes, but rather, was in understanding the information conveyed there.

Beyond establishing that older adults have difficulty interpreting eyes information, a crucial question is, why? Previous researchers have appealed to brain decline. Although we did not examine brain decline in our study, we think it is essential to consider this argument when trying to explain our findings. Thus, some have argued that decline in the orbitofrontal cortex, amygdala and anterior cingulate cortex are likely causes of older adults’ difficulty recognizing anger, sadness and fear^2,19^, and that decline in the orbitofrontal cortex, amygdala and superior temporal sulcus are likely causes of difficulty interpreting eyes information^10^. The orbitofrontal cortex and anterior cingulate cortex are part of the ventromedial prefrontal cortex (vmPFC) region, so vmPFC decline is potentially central to both emotion recognition decline and difficulty decoding eyes information.

In contrast, some argue that age differences in emotion recognition “are better explained by factors other than age-related vmPFC decline”, in particular, by decline in the dorsolateral prefrontal cortex, which is associated with working memory and a failure to “attend to the eyes”^18^. However, for the reasons given above, attention to the eyes would not seem to be the problem; when we presented the eyes alone, with nowhere else to look, older adults were still worse recognizing anger, sadness and fear in the eyes. In contrast, the vmPFC declines relatively rapidly with age^2,19^, which would explain the difficulties recognizing anger, sadness and fear in eyes and faces, and less looking at the eyes. Furthermore, it is worth reiterating that inattention to the eyes cannot explain older adults’ broader difficulties decoding emotion from voices or bodies, whereas decline in the vmPFC can.

### Culture

As in many studies, we found a significant emotion recognition advantage for same-race stimuli, indicating that our stimuli were comparable to those used in previous studies. Nevertheless, there was a striking difference in performance between Easterners and Westerners despite us using both Eastern and Western stimuli. Whereas there was no difference in the performance of Easterners and Westerners on any of the six emotions when given the eyes only (0/6 comparisons), Easterners were worse on all six emotions when given the mouth only or full face (12/12 comparisons). Our findings provide the clearest indication that Easterners benefit most from eyes information (where they are equal to Westerners), but often struggle with mouths on their own or full faces. Recall that previous research, which examined correlations between recognition and Easterners’/Westerners’ gaze resulted in conflicting findings in this regard, whereas our findings provide very clear evidence for Easterners’ relative strength on eyes but difficulty with mouths and full faces. This finding, clearly indicating Easterners’ pronounced difficulty with mouths but not eyes, is entirely novel.

Thus, our results help to qualify the findings of previous researchers who have found lower emotion recognition in Eastern compared to Western individuals^22^. First, as stated, Easterners are *not* worse when just the eyes are presented. Second, when Easterners do have problems on full faces, their difficulty with mouths helps to explain why, given that mouths are generally more informative than eyes (see the main effect for Emotion above in which emotion recognition was better with mouths only than with eyes only).

Our findings also help to explain the results of a recent study that indicated Easterners tend to have three categories of emotion faces: happy, sad/angry/disgusted, and fearful/surprised^36^. Happy expressions can be distinguished uniquely by an upturned mouth, and despite Easterners having some difficulty decoding mouth information, happy mouths are different from all other emotions and are thus easier to decode. However, because Easterners have an over-reliance on eyes, and fearful and surprised eyes are very similar (open eyes, eyebrows raised) and differ only in the mouth (surprise: open mouth; fear: mouth pulled back at the lip corners), they have difficulty differentiating fear and surprise. Likewise, sad, angry and disgusted expressions are similar in that they all involve contraction of the corrugator muscle between the eyes^37,38^ but differ in the mouth muscles contracted, with anger involving contraction of AU23 (lip tightener), sadness involving contraction of AU15 (lip corner depressor), and disgust involving contraction of AU15 and AU17 (chin raiser)^37^.

Thus, the lingering question is, why would Easterners struggle to identify mouth information? First, two potential reasons can likely be ruled out. Easterners’ difficulty cannot be due solely to an inability to process stimuli holistically (i.e., with whole-face stimuli) given that they had difficulty with the mouth on its own in addition to the whole face. Nor can language difficulties account for the results given that all participants spoke good English, and there were no difficulties for Singapore participants on the Eyes Only stimuli. Instead, there might be several reasons. First, Easterners tend to express emotions less intensely than Westerners^39,40^, which could hinder general learning about how emotions are expressed. Asian cultures are predominantly influenced by Confucianism^41^, which argues for suppressing public displays of emotion^42^. Even loud and hearty laughter is deemed undesirable as it is thought to reflect ill-breeding. Nevertheless, although these tendencies might suppress emotion recognition generally, they don’t explain Easterners’ ability with eyes or difficulties with full faces and mouths, so there must be an explanation that is tied more to mouths in particular. More specifically, etiquette dictates that Easterners, and women in particular, do not bare their teeth or open their mouths wide when laughing^43^. As a result, Easterners instinctively cover their mouths when laughing, which would result in fewer opportunities for observers to learn to identify emotions in mouths, and more difficulty recognizing emotions from mouths or full faces. In contrast, opportunities to learn about expressions in eyes would be unaffected.

### Possible objections to presenting just the eyes or just the mouth

In our pilot work, we have tried to present full faces but instruct participants to look at either the eyes or the mouth. Despite this instruction, the eye-tracking data indicated that participants often look at the non-target face region, and since emotion recognition is relatively good with only 1/15th of a second exposure-time^44^, this method makes it impossible to ensure that emotion recognition is based only on the target facial region. Furthermore, even if a participant could refrain from looking at the non-target face region, extra-foveal processing (peripheral vision) also contributes to emotion recognition, and so again, the non-target region would contribute to recognition^45^. A second method that has been used to focus processing on a particular face region is to present faces briefly (e.g., 80 ms), with the time insufficient for a saccade to occur to a non-target area^45^. There are two difficulties with employing this method in the present study. First, processing speed reliably slows with age^46^, making a fixed presentation time either too long for young adults (so that they look elsewhere and fixations are not restricted to the target area) or too short for older adults (so that they stay fixated on the target area but their emotion recognition is impaired due to insufficient processing time). Second, enforcing fixation to a particular region, when the whole face is present, would still allow extra-foveal processing as described above, and would defeat the purpose of ensuring that only one face region was used to infer emotion. Thus, to ensure that emotion processing was restricted to just the eyes or just the mouth, it was essential to present just the eyes or just the mouth, with this manipulation yielding highly novel information about the varied nature of the emotion recognition strengths and difficulties of older adults and Asian individuals.

It might also be argued that poor visual acuity stifled older adults’ emotion recognition, for instance, due to difficulty perceiving either low or high frequency spatial information. Yet, this would not explain why older adults were significantly worse on four emotions from the eyes, but only one from the mouth. The spatial frequencies of the photographs used for eyes only, mouth only or the full face were identical. Likewise, it would not explain why Easterners found recognition from mouths and the full face much more difficult than from the eyes only. Thus, there is no evidence that spatial frequencies or visual acuity difficulties led to the pattern of results we obtained.

## Conclusion

Previous research demonstrated that older adults and Easterners sometimes struggle to identify emotions in faces. Our findings provide critical information as to when this is true. Older adults are frequently worse recognizing emotions in the eyes as has sometimes been hypothesized, and are less frequently worse when recognizing emotion in mouths. There were also striking cross-cultural effects with Easterners and Westerners equal when decoding the eyes, but Easterners much less skilled when decoding mouths or full faces.

## Methods

### Participants

The Western participants were recruited from those of European descent residing in a small city in New Zealand, and the Easterners from those of Asian Chinese descent living in Singapore. There were 54 young Westerners (*M*_age_ = 19.52 years, range = 17–26, 28 women), 54 young Easterners (*M*_age_ = 21.33 years, range = 19–31, 28 women), 56 older Westerners (*M*_age_ = 71.48 years, range = 60–90, 27 women), and 53 older Easterners (*M*_age_ = 68.19 years, range = 60–85, 27 women). The sample size was based on an a priori power analysis using G*Power^47^, with a projected effect size of 0.25 for all effects. This effect is regarded as a “medium” effect size, which is what is typically reported in prior studies of emotion recognition. Table S3 in the Supplemental Materials contains more information about power. These calculations established that a sample of 128 participants is sufficient to detect a medium-sized effect in a 2-way interaction, but we over-sampled to 200 participants.

Within New Zealand, young adults were recruited from the Psychology Department and were given course credit for their participation, and older adults were recruited from referrals, personal contacts and our own database. Within Singapore, young adults from the National University of Singapore participated in the study and were given university course credit for their participation, and 53 older adults were recruited via referrals and personal contacts. Older adults were given travel money. All participants were competent English speakers, were mentally sound without a history of stroke, and had normal or corrected-to-normal vision.

All older adults were also screened using the Mini-Mental State Examination (MMSE)^48^ and only scores greater than 25 out of 30 were included in the dataset.

### Materials

There were 108 colour emotion photographs of young adults on a white background (36 Full Face, 36 Eyes Only, 36 Mouth Only). The Eyes Only and Mouth Only photographs were taken from the Full Face photographs. Half of the faces were Western European^49^ and half were Eastern Chinese^50^. The Western faces have been used in previous studies of age differences in emotion recognition^3^. The Eastern face database was normed^49^, and used in subsequent research^36,51,52^. Each stimulus was presented on a monitor that was 1920 × 1080 pixels in resolution. For both the Eastern and Western faces, half were male and half were female. The 36 Full Face pictures (six each for angry, sad, fearful, disgusted, surprised and happy) were against a light-coloured background, with faces spanning vertically from hairline (with foreheads exposed) to the chin, and horizontally between the left and right ear. For the Eyes Only and Mouth Only pictures, truncation was made from the bridge of the nose to the upper or lower part of the face. The faces and six emotion labels remained present until the participant responded.

### Procedure and design

There were no time constraints during the experiment. We gave participants the three sets of emotion items (Full Face, Eyes Only and Mouth Only stimuli), with sets and items within each set, given in a random order. For the younger adults, testing was conducted at the Psychology laboratory. Testing for the older adults in both Singapore and New Zealand was conducted in participants’ preferred venues (typically their homes or offices) given that they were not on-campus like the young adults, this was their preferred place of testing, and research indicates no difference in results for in-lab versus at-home cognitive testing for older adults^53^. The same researcher conducted all testing, using the same procedure and apparatus, and with testing in English given that all participants were competent English speakers.

To gauge emotion recognition and avoid potentially biased responses towards certain emotions, we used the unbiased hitrate (H_u_) proposed by Wagner (1993). H_u_ was calculated as the squared frequency of accurate responses for a targeted emotion divided by the product of the overall number of stimuli and the total frequency of the emotion examined. Hu ranged from 0 to 1, 0 being the emotion that was falsely selected, and 1 indicating emotion recognition accuracy. We then tabulated Hu for each participant, each emotion, and each face region (Full Face, Eyes Only and Mouths Only) across gender, age and ethnic group.

### Ethics and additional considerations

Ethical approval for the study was obtained by the respective governing bodies of both countries (New Zealand: University Human Ethics Panel, #F17/008; National University of Singapore IRB: 2018-November-124). The study was carried out consistent with American Psychological Association ethical guidelines. Prior to beginning, informed consent was obtained from all participants. We also collected eye tracking data for an additional task in which participants viewed full emotion faces after all stimuli in the present study had been administered, but for the sake of brevity, do not report these results. The data and stimuli are available from the corresponding author. We did not pre-register this study.

## Supplementary Information

Supplementary Tables.
