# Supplementary material for: Older adults have difficulty decoding emotions from the eyes, whereas easterners have difficulty decoding emotion from the mouth
Source: Sci Rep. 2022 May 6;12:7408. doi: 10.1038/s41598-022-11381-8 (PMC9076610; doi:10.1038/s41598-022-11381-8)
Supplement: Supplementary file 1 — Supplementary Tables. [file 41598_2022_11381_MOESM1_ESM.docx]

**Supplementary Materials**

**Table S1**

*Confusion Matrices for Young and Older Adults on Full Face Stimuli*

|  |  | Percentage Response Given | | | | | |
| --- | --- | --- | --- | --- | --- | --- | --- |
|  |  | Anger | Sadness | Fear | Disgust | Surprise | Happiness |
| **Young**  Correct Response | Anger | 92.7 | 3.4 | 0.3 | 3.1 | 0.2 | 0.3 |
|  | Sadness | 0.8 | 86.0 | 3.1 | 8.6 | 1.5 | 0 |
|  | Fear | 0.4 | 4.3 | 69.8 | 10.7 | 14.8 | 0 |
|  | Disgust | 13.6 | 1.9 | 0.3 | 81.6 | 0.6 | 2.0 |
|  | Surprise | 0.1 | 0.4 | 14.7 | 1.2 | 82.1 | 1.5 |
|  | Happiness | 0 | 0.8 | 0.3 | 0 | 0.2 | 98.8 |
| **Older**  Correct Response | Anger | 85.0 | 2.3 | 0.6 | 11.2 | 0.9 | 0 |
|  | Sadness | 2.6 | 78.0 | 6.6 | 10.2 | 1.8 | 0.8 |
|  | Fear | 3.7 | 3.5 | 65.3 | 5.7 | 21.4 | 0.4 |
|  | Disgust | 11.9 | 3.2 | 2.9 | 76.0 | 4.3 | 1.7 |
|  | Surprise | 0.4 | 1.2 | 14.3 | 1.7 | 80.6 | 1.8 |
|  | Happiness | 0 | 0.3 | 0 | 0.2 | 1.1 | 98.5 |
| Young Adult | Mean Errors % | 16.1 | 10.9 | 21.3 | 27.0 | 20.1 | 4.6 |
| Older Adult | Mean Errors % | 15.6 | 8.9 | 21.3 | 25.8 | 25.3 | 3.1 |

*Note*. The final two rows of the table indicate the percentage of errors that were angry, sad, fearful, disgusted, surprised or happy for each group.

**Table S2**

*Confusion Matrices for Easterners and Westerners on Full Face Stimuli*

|  |  | Percentage Response Given | | | | | |
| --- | --- | --- | --- | --- | --- | --- | --- |
|  |  | Anger | Sadness | Fear | Disgust | Surprise | Happiness |
| **Easterners**  Correct Response | Anger | 90.0 | 1.4 | 0.3 | 7.3 | 1.1 | 0.2 |
|  | Sadness | 1.4 | 85.0 | 3.3 | 8.6 | 1.4 | 0.3 |
|  | Fear | 2.8 | 4.3 | 60.0 | 13.1 | 19.6 | 0.2 |
|  | Disgust | 16.7 | 3.4 | 2.2 | 72.1 | 2.5 | 2.3 |
|  | Surprise | 0.3 | 0.3 | 11.5 | 2.1 | 83.0 | 2.5 |
|  | Happiness | 0 | 0.8 | 0.2 | 0.2 | 0.8 | 98.1 |
| **Westerners**  Correct Response | Anger | 87.7 | 4.2 | 0.6 | 6.8 | 0.5 | 0.2 |
|  | Sadness | 2.0 | 78.9 | 6.4 | 10.3 | 2.0 | 0.5 |
|  | Fear | 1.3 | 3.5 | 74.9 | 3.5 | 16.7 | 0.2 |
|  | Disgust | 8.2 | 1.7 | 1.0 | 85.3 | 2.4 | 1.4 |
|  | Surprise | 0.3 | 1.3 | 17.1 | 0.8 | 79.7 | 0.8 |
|  | Happiness | 0 | 0.3 | 0.2 | 0 | 0.5 | 99.1 |
| Easterners | Mean Errors % | 19.0 | 9.0 | 15.8 | 28.5 | 22.6 | 5.0 |
| Westerners | Mean Errors % | 13.0 | 12.5 | 23.4 | 23.9 | 23.9 | 3.3 |

*Note*. The final two rows of the table indicate the percentage of errors that were angry, sad, fearful, disgusted, surprised or happy for each group.

**Table S3**

*Power Analyses*

| **Analysis** | Test Family | Stat Test | Type | Effect Size | *α* | Power | # Groups | # Measures | Correla. Among Measures | Total Sample Size |
| --- | --- | --- | --- | --- | --- | --- | --- | --- | --- | --- |
| Participant Ethnicity x Participant Age: main effects and interaction | *F* tests | 1 | * | .25 (Medium) | .05 | .80 | 2 | 4 | NA | 128 |
| Participant Age/Ethnicity x Face Region: Between-Subjects Variable | *F* tests | 2 | * | .25 (Medium) | .05 | .80 | 2 | 3 | .5 | 86 |
| Participant Age/Ethnicity x Face Region: Within-Subjects Variable | *F* tests | 2 | * | .25 (Medium) | .05 | .80 | 2 | 3 | .5 | 28 |
| Participant Age/Ethnicity x Face Region: interaction | *F* tests | 2 | * | .25 (Medium) | .05 | .80 | 2 | 3 | .5 | 28 |

*Note*. *: All tests are a priori. 1: Between-subjects, two factors. 2: One between-subjects factor, one within-subjects factor. NA: not applicable. For the last three rows of the table, power statistics are shown for when the between subjects-variable is either Participant Age or Participant Ethnicity and when the within-subjects variable is Face Region. The number of participants needed when Emotion is the dependent variable is always less if this is the dependent variable rather than Face Region.
